# Reducing dietary crude protein for gestating and lactating sows reduces daily nitrogen retention, but reproductive performance is not impacted by diet protein concentration

**DOI:** 10.1186/s40104-026-01445-4

**Published:** 2026-06-19

**Authors:** Jimena A. Ibagon, Su A Lee, Hans H. Stein

**Affiliations:** Department of Animal Sciences, University of Illinois, Urbana, IL 61801 USA

**Keywords:** Crystalline amino acids, Nitrogen balance, Reproductive performance, Sows, Soybean meal

## Abstract

**Background:**

Soybean meal (SBM) is a primary protein source in swine diets, but partial replacement with crystalline amino acids (AA) is commonly used to reduce dietary protein. Adequate dietary protein and energy are essential to support fetal development, milk production, and litter growth. However, crystalline AA are absorbed more rapidly than AA from intact protein, which may limit protein synthesis due to a lack of AA availability. Therefore, an experiment was conducted to test the hypothesis that feeding sows diets based primarily on corn, SBM, and no crystalline AA will result in improved reproductive performance and immunity of sows compared with sows fed diets with less SBM and more corn and crystalline AA.

**Results:**

Nitrogen excretion in feces and urine, absorbed nitrogen, and retained nitrogen (g/d) were greater (*P* < 0.05) in gestating sows fed the high-protein diet compared with sows fed the low-protein diet. Rectal temperature 24 h after farrowing of sows fed the low-protein diet was greater (*P* < 0.05) compared with sows fed the high-protein diet. Number of live-born and total born pigs was not different between treatments, but sows fed the high-protein diet tended to produce fewer (*P* < 0.10) mummified pigs than sows fed the low-protein diet. Malondialdehyde was greater (*P* < 0.05) in sows fed the low-protein diet, but serum glutathione peroxidase and white blood cell count were greater (*P* < 0.05) in sows fed the high-protein diet. Colostrum immunoglobulin G and concentrations of fat, protein, urea nitrogen, lactose, and immunoglobulin G were greater (*P* < 0.001) in milk from sows fed the high-protein diet than in milk from sows fed the low-protein diet.

**Conclusions:**

Feeding a low-protein diet to gestating sows decreased daily nitrogen retention. Reproductive performance was not affected, but feeding a high-protein diet without crystalline AA resulted in greater concentrations of fat, protein, and lactose in milk and improved immune-related characteristics compared with feeding a low-protein diet.

## Background

Soybean meal (SBM) is an important protein source that is often used to furnish the majority of amino acids (AA) in diets for sows, but reduction in SBM and inclusion of crystalline AA may sometimes reduce diet costs [1]. Over the past few decades, reproductive efficiency in sows has improved due to genetic selection, resulting in litters that can exceed 20 pigs [2]. This increase in prolificacy has increased the metabolic demands for AA and energy during both gestation and lactation [2–4]. Adequate energy intake in gestation supports fetal growth and mammary development [5], but excessive energy intake during gestation may reduce feed intake during lactation, which has a negative impact on milk production [6]. However, high protein intake during gestation improves milk production, protein accretion, and litter and pig weight at weaning [7], but it may also result in an increase in fat accretion because greater nutrient supply promotes both protein and lipid deposition in gestating sows [8].

Although crystalline AA may support growth performance, protein synthesis can only occur when all required AA are simultaneously available in the cell [9]. Crystalline AA may be absorbed more rapidly than AA from intact proteins, which may result in early oxidation of crystalline AA before AA from intact protein arrives in the cell, limiting protein synthesis [10, 11]. It is, therefore, unknown, if low-protein diets containing crystalline AA can support the same reproductive performance, litter growth, and immune status of sows as feeding diets that are based on only corn and SBM and no crystalline AA. An experiment was, therefore, conducted to test the hypothesis that feeding sows diets based primarily on corn and SBM, and with no crystalline AA may result in improved reproductive performance and immunity of sows compared with sows fed diets with less SBM and more corn and crystalline AA.

## Methods

The Institutional Animal Care and Use Committee at the University of Illinois reviewed and approved the protocol for the experiment before animal work was initiated. The experiment was conducted at the Swine Research Center at the University of Illinois at Urbana-Champaign (IL, USA) from August 2023 to May 2024.

### Experimental diets

Two gestation diets and two lactation diets were formulated to meet estimated requirements for AA and other nutrients by gestating and lactating sows [12] (Table 1). Within each phase of production, one diet was a high-protein diet in which AA were furnished by corn and SBM, and the other diet was a low-protein diet in which the inclusion of SBM was reduced and crystalline AA were included to meet requirements for gestating or lactating sows. Both gestation diets also contained soybean hulls. The high-protein gestation and lactation diets contained 17.00% and 24.34% SBM, respectively, whereas the low-protein gestation diet contained 7.65% SBM, and the low-protein lactation diet contained 12.60% SBM. All diets were fed as mash diets. Twelve batches of gestation diets and seven batches of lactation diets were mixed during the experiment. Diet samples were collected from each batch, and at the conclusion of the experiment, diet samples were pooled and subsampled for chemical analysis. Ingredient samples were also collected, pooled, and subsampled for analysis.

**Table 1:** Ingredient and nutrient compositions of experimental diets (as-fed basis)

Item	Gestation		Lactation	
	High protein	Low protein	High protein	Low protein
Ingredient, %				
Corn	68.89	77.61	70.780	81.65
Soybean meal	17.00	7.65	24.34	12.60
Soybean hulls	10.00	10.00	-	-
Soybean oil	1.00	1.00	2.00	2.00
L-Lys·HCl	-	0.29	-	0.37
DL-Met	-	0.06	-	0.02
L-Thr	-	0.12	-	0.12
L-Trp	-	0.03	-	0.04
L-Val	-	-	-	0.15
Dicalcium phosphate	1.42	1.58	1.28	1.47
Calcium carbonate	0.79	0.76	0.70	0.67
Sodium chloride	0.40	0.40	0.40	0.40
Vitamin-mineral premix^1^	0.50	0.50	0.50	0.50
Analyzed nutrients				
Dry matter, %	85.34	84.66	84.46	83.76
Ash, %	4.16	4.17	2.11	2.21
Crude protein, %	14.14	11.19	16.14	12.60
Gross energy, kcal/kg	3,901	3,808	3,890	3,832
Indispensable amino acids, %				
Arg	0.79	0.55	0.98	0.65
His	0.36	0.28	0.45	0.31
Ile	0.58	0.42	0.72	0.49
Leu	1.28	1.01	1.48	1.16
Lys	0.74	0.73	0.87	0.83
Met	0.22	0.21	0.25	0.22
Phe	0.69	0.51	0.82	0.59
Thr	0.51	0.48	0.60	0.55
Trp	0.12	0.10	0.18	0.13
Val	0.68	0.51	0.80	0.72
Total	5.97	4.80	7.15	5.65
Indispensable amino acids as % of total	44.79	46.33	45.77	47.80
Dispensable amino acids, %				
Ala	0.76	0.62	0.85	0.69
Asp	1.30	0.90	1.55	1.02
Cys	0.24	0.19	0.26	0.22
Glu	2.50	1.85	2.96	2.11
Gly	0.61	0.47	0.66	0.46
Pro	0.88	0.72	0.97	0.79
Ser	0.60	0.47	0.68	0.51
Tyr	0.47	0.34	0.54	0.37
Total	7.36	5.56	8.47	6.17
Dispensable amino acids as % of total	55.21	53.67	54.23	52.20
Total amino acids, %	13.33	10.36	15.62	11.82

^1^The vitamin-micromineral premix provided the following quantities of vitamins and micro minerals per kg of complete diet: vitamin A as retinyl acetate, 10,622 IU; vitamin D_3_ as cholecalciferol, 1,660 IU; vitamin E as DL-alpha-tocopherol acetate, 66 IU; vitamin K as menadione nicotinamide bisulfate, 1.40 mg; thiamin as thiamine mononitrate, 1.08 mg; riboflavin, 6.49 mg; pyridoxine as pyridoxine hydrochloride, 0.98 mg; vitamin B_12_, 0.03 mg; D-pantothenic acid as D-calcium pantothenate, 23.2 mg; niacin, 43.4 mg; folic acid, 1.56 mg; biotin, 0.44 mg; Cu, 20 mg as copper chloride; Fe, 123 mg as iron sulfate; I, 1.24 mg as ethylenediamine dihydroiodide; Mn, 59.4 mg as manganese hydroxychloride; Se, 0.27 mg as sodium selenite and selenium yeast; and Zn, 124.7 mg as zinc hydroxychloride

### Animals, housing, and feeding

#### Gestation housing and feeding

A total of 154 Camborough gilts and sows (Pig Improvement Company, Hendersonville, TN, USA) were bred to terminal line boars (Pig Improvement Company L 800). The initial body weight was 190.0 ± 26.8 kg. Sows and gilts were used in eight blocks of 20 to 28 animals, using a randomized complete block design. The breeding group was the blocking factor. Within each block, animals were allotted to experimental diets on the day of breeding, with parity balanced between treatments, and feeding of gestation diets started on the day of breeding and continued until d 104 of gestation. At this time, sows were moved to the lactation facility, and feeding of the lactation diets was initiated. During gestation, sows were housed individually in gestation stalls (2.10 m × 0.60 m). During the gestation period, daily feed allotments were provided at 0600 h. Feed allowance was 1.5 times the maintenance requirement for metabolizable energy for gestating sows (i.e., 100 kcal metabolizable energy/kg body weight^0.60^; [12]), but feed allowance was adjusted every other week, to maintain or achieve an ideal sow body condition by visual scoring (approximately 3.0 on a 1 to 5-point scale; [13]).

#### Nitrogen balance

From the 154 animals that were initially assigned to the experimental diets, 90 sows (parity 2 to 6) were placed in individual metabolism crates (0.91 m × 2.08 m) from d 45 to 56 (i.e., mid-gestation). Within each block, sows were evenly distributed between the two treatments and with the same parity, with 12 sows in blocks one through six (i.e., six sows per treatment) and nine sows in blocks seven and eight (i.e., four or five sows per treatment), for a total of 44 and 46 sows for the high-protein and low-protein diets, respectively. Crates were equipped with a self-feeder, a nipple waterer, and a fully slatted floor to allow for total, but separate, collection of urine and fecal materials. The selected 90 sows had an average parity of 3.4 ± 1.3, and an average body weight of 200.4 ± 18.5 kg when moved to the metabolism crates. A screen floor was installed under the slatted floor, and feces were quantitatively collected from the screen floor. A urine tray was installed under the screen floor, and urine was captured in this tray and drained into a urine bucket that was placed under the tray, which allowed for quantitative collection of urine. The initial 3 d in the metabolism crates were considered the adaptation period, whereas urine and fecal materials were collected from feed provided during the following 5 d according to standard procedures using the marker-to-marker approach [14]. Fecal collection was initiated when the first marker (i.e., indigo carmine) appeared in the feces and ceased when the second marker (i.e., ferric oxide) appeared [14]. After the 5 d of collection, sows stayed in the metabolism crates for three additional d until appearance of the second marker was confirmed. To avoid nitrogen loss in the urine, 50 mL of 6 mol/L HCl was added to the urine bucket daily. Buckets were emptied daily, and the weight of the collected urine was recorded, and 10% was stored at −20 °C until subsampling. Orts were collected daily prior to feeding the morning meal, pooled for the duration of the collection period, dried in a 65 °C forced-air drying oven (Thermo Fisher Scientific Inc.; model Heratherm OMH750, Waltham, MA, USA), and weighed to determine feed intake during the collection period. Fecal samples from each animal were stored at −20 °C immediately after collection. Urine samples were thawed and mixed within animal and diet at the conclusion of the experiment, and a subsample was stored for nitrogen analysis. Fecal samples from each sow were thawed and mixed, and then dried in a 65 °C forced-air drying oven as described for orts. All dried fecal samples were finely ground using a 500 G stainless steel swing-type mill grinder (RRH, Zhejiang, China), and the ground samples were mixed, and a subsample was collected for chemical analysis.

#### Lactation housing, feeding, and performance

Sows were moved to the lactation unit on d 104 of gestation and housed individually in farrowing crates. Each farrowing crate (2.10 m × 1.50 m) was equipped with a stainless-steel feeder and two nipple waterers. Sows were fed experimental lactation diets starting the day they were moved to the lactation unit. Sows were fed as in gestation from entry to the farrowing unit until farrowing, but diets were provided on an ad libitum basis from farrowing until weaning and water was available at all times. According to normal farm procedures, all litters were offered a standard creep diet from d 14 post-farrowing until weaning.

Sow body weights were determined on the day of breeding, when sows were moved in and out of metabolism crates, when sows were moved to the lactation barn, within 24 h after farrowing, and on the day of weaning. Weaning took place at 20.39 ± 0.71 d post-farrowing. On the day of farrowing and 24 h later, the rectal temperature of each sow was measured using a digital thermometer while no feeding or nursing activity took place.

The number and body weight of pigs born alive, the number of mummies, stillborn pigs, and total pigs per litter after cross-fostering were recorded, and pigs were weighed again at weaning. For both dietary treatments, cross-fostering was completed within 24 h of farrowing and pigs were only cross-fostered within treatment group, and each sow had approximately 14 pigs after cross-fostering. Following normal farm procedures, pigs weighing less than 0.9 kg at birth were considered low vitality and euthanized. The weight of pigs that died during lactation, as well as the reason for death (crushed by sow, low vitality/starved, rupture, or euthanized due to congenital deformity), were recorded. Pigs were processed within 24 h of birth. Processing included clipping needle teeth, docking tails, castrating male pigs, administering iron dextran (Uniferon, Pharmacosmos, Watchung, NJ, USA), and ceftiofur antibiotic (Excede, Zoetis, Parsippany, NJ, USA), and also ear notching for identification.

### Blood and milk sample collection

Blood samples were collected from all sows 14 d post-farrowing. Two blood samples were collected from the jugular vein via venipuncture. One blood sample was collected in vacutainers with ethylenediaminetetraacetic acid, and the other blood sample was collected in serum vacutainer tubes containing spray-coated silica as a serum clot activator. Blood samples were stored on ice immediately after collection, and ethylenediaminetetraacetic acid blood samples were delivered to the University of Illinois Veterinary Diagnostic Laboratory (Urbana, IL, USA) for analysis of white blood cells, neutrophil, and lymphocyte cell counts in the whole blood. The blood collected from the serum vacutainer tubes was allowed to clot and then centrifuged at 1,000 × *g* for 10 min at room temperature. Serum was removed from centrifuged tubes and stored at −80 °C until analysis. Colostrum samples were collected within 24 h of farrowing, and milk samples were collected on d 14 post-farrowing following administration of 1 mL oxytocin (Bimeda-MTC Animal Health Inc., Cambridge, ON, Canada) intramuscularly. Approximately 70 mL of colostrum or milk was collected in 50 and 25 mL conical sterile polypropylene centrifuge tubes from the first five functional teats on each side of the mammary gland. Colostrum and milk samples were stored at −20 °C immediately after collection. Prior to shipping milk for component analysis, all samples were thawed, placed in 60-mL tubes containing a milk preservative, and a subsample of 10 mL was placed in a separate tube for later analysis.

### Chemical analyses

Ingredients, diets, and fecal samples were analyzed for dry matter by oven drying at 135 °C for 2 h (method 930.15; [15]) and for dry ash (method 942.05; [15]). The concentration of nitrogen in diets and ingredients was analyzed using the Kjeldahl method (method 984.13; [15]) on a Kjeltec™ 8400 (FOSS Inc., Eden Prairie, MN, USA) with subsequent calculation of crude protein using a conversion factor of 6.25. These samples were also analyzed for AA on a Hitachi Amino Acid Analyzer (Model No. L8800; Hitachi High Technologies America, Inc., Pleasanton, CA, USA) using ninhydrin for postcolumn derivatization and norleucine as the internal standard. Prior to analysis, samples were hydrolyzed with 6 mol/L HCl for 24 h at 110 °C (method 982.30 E(a); [15]). Methionine and Cys were determined as Met sulfone and cysteic acid after cold performic acid oxidation overnight before hydrolysis (method 982.30 E(b); [15]). Tryptophan was determined after NaOH hydrolysis for 22 h at 110 °C (method 982.30 E(c); [15]). Ingredients were analyzed for phytic acid [16], and total starch was determined using the amyloglucosidase-alpha-amylase procedure corresponding to the enzymatically hydrolyzed starch converted to glucose, followed by analysis of the glucose concentration by spectroscopy (method 996.11; [15]), whereas glucose, sucrose, maltose, fructose, stachyose, and raffinose were analyzed using high-performance liquid chromatography (method 977.2; [15]). Acid hydrolyzed ether extract was also analyzed by acid hydrolysis using 3 mol/L HCl (Ankom HCl Hydrolysis System, Ankom Technology, Macedon, NY, USA), followed by fat extraction (Ankom XT-15 Extractor, Ankom Technology, Macedon, NY, USA). Insoluble dietary fiber and soluble dietary fiber were also analyzed in ingredients according to method 991.43 [15] using the Ankom Dietary Fiber Analyzer (Ankom Technology). Total dietary fiber was calculated as the sum of insoluble dietary fiber and soluble dietary fiber. Soybean meal and soybean hulls were also analyzed for trypsin inhibitors (method Ba 12-75; [17]). Fecal and urine samples were analyzed for nitrogen as described for diets. Milk samples were analyzed by Eastern Laboratory Services (Medina, OH, USA) for fat, free fatty acids, protein, milk urea nitrogen (MUN), lactose, other solids, total solids, and somatic cell count (SCC) using a Milkoscan 7 calibrated for bovine milk (Foss, Hillerød, Denmark), and colostrum samples were analyzed for protein, fat, and total solids. Concentrations of interleukin (IL)-1α, IL-1β, IL-1 receptor antagonist, IL-2, IL-4, IL-6, IL-8, IL-10, IL-12, IL-18, interferon-γ (IFNγ), and tumor necrosis factor-α (TNF-α) in serum samples were measured using a porcine-specific multiplex immunoassay kit (MilliporeSigma, Burlington, MA, USA) and read with a Luminex MagPix instrument (Luminex Corporation, Austin, TX, USA). Malonaldehyde and glutathione peroxidase in serum samples were determined by enzyme-linked immunosorbent assay following the manufacturer’s instructions (MyBioSource, Inc., San Diego, CA, USA). Immunoglobulin G (IgG) in milk and colostrum was also determined by enzyme-linked immunosorbent assay following the manufacturer’s instructions (Bethyl Laboratories, Inc., Montgomery, TX, USA).

### Calculations and statistical analyses

At the conclusion of the gestation period, the apparent total tract digestibility (ATTD) of dry matter and nitrogen, retention of nitrogen, and biological value of nitrogen were calculated [12, 18]. Data for body weight gain in gestation, body weight loss in lactation, average daily feed intake (ADFI) in gestation and lactation, estimated milk yield (calculated as 4 g milk per g of litter body weight gain; [19]), and litter performance data were calculated. Litter performance data were calculated for each sow and included number of total pigs born, live born pigs, mummified pigs, and still born pigs; number of pigs after cross-fostering; number of pigs weaned; and pig mortality rates (calculated as the percentage of live born pigs that died before weaning before and after adjusting for cross-fostering). Total live litter birth weight, litter birth weight after cross fostering, litter weight at weaning, and litter average daily gain (ADG) were calculated as well. Average pig weights and ADG were also calculated.

Model assumptions on the residuals were confirmed using the MIXED procedure and the Brown-Forsythe test of the GLM procedure of SAS (SAS Inst. Inc., Cary, NC, USA). The MIXED procedure of SAS was used to generate studentized residuals, and outliers were defined as observations having residuals greater than 3 or less than −3. A sow was excluded from statistical analysis if three or more response variables were identified as outliers, but of the 154 sows that farrowed, only one sow from the high-protein diet was identified as an outlier and excluded from statistical analyses. All other sows were included in the final analysis. Except for the number of pigs born alive, the number of mummies, stillborn pigs, and total pigs born per litter, data were considered continuous variables and analyzed using the MIXED procedure in SAS. The number of pigs born alive, mummified pigs, stillborn pigs, and the total pigs born per litter were considered discrete count variables and analyzed using the GLIMMIX procedure in SAS. Mortality data were considered binomial variables and were also analyzed using the GLIMMIX procedure in SAS. The initial statistical model for the MIXED and the GLIMMIX procedures included the fixed effects of diet, and block and replicate within block as random effects. However, for litter performance response variables, litter weight after cross-fostering was included as a covariate to account for variation in litter weight. The sow was the experimental unit for all sow-related variables, whereas the litter was the experimental unit for litter performance variables. Least square means for somatic cell count and cytokines were reported in the original scale after back-transforming (inverse log) the output from the LSMEANS statement in the MIXED procedure. The LSMEANS statement was used to calculate treatment means for all other variables in the MIXED procedure, along with the inverse link option in the GLIMMIX procedure. Results were considered significant at *P* ≤ 0.05 and considered a tendency at 0.05 < *P* ≤ 0.10.

## Results

The chemical compositions of the diets and the ingredients were, in general, in agreement with expected values (Tables 1 and 2).

**Table 2:** Analyzed composition (as-is basis) of ingredients used in gestation and lactation diets

Item	Corn	Soybean meal	Soybean hulls
Dry matter, %	87.86	89.66	90.01
Ash, %	1.26	6.41	4.55
Crude protein, %	8.01	47.10	10.99
Acid hydrolyzed ether extract, %	3.68	2.81	1.80
Total dietary fiber, %	12.10	18.30	66.70
Insoluble dietary fiber, %	11.50	16.30	62.90
Soluble dietary fiber, %	0.60	2.00	3.80
Phytic acid, %	0.63	1.59	0.16
Starch, %	62.30	0.96	0.96
Glucose, %	0.32	0.05	0.19
Maltose, %	0.52	0.38	0.05
Fructose, %	0.07	0.08	0.14
Sucrose, %	0.52	6.50	0.45
Stachyose, %	0.08	5.44	0.64
Raffinose, %	0.09	1.11	0.16
Trypsin inhibitors, units/mg	-	0.04	4.53
Indispensable amino acids, %			
Arg	0.34	3.10	0.59
His	0.21	1.14	0.28
Ile	0.28	2.06	0.51
Leu	0.89	3.36	0.85
Lys	0.24	2.83	0.78
Met	0.16	0.65	0.13
Phe	0.38	2.26	0.47
Thr	0.28	1.75	0.47
Trp	0.05	0.60	0.06
Val	0.37	2.19	0.55
Dispensable amino acids, %			
Ala	0.56	1.90	0.52
Asp	0.55	5.00	1.21
Cys	0.17	0.66	0.25
Glu	1.45	8.03	1.42
Gly	0.31	1.84	0.91
Pro	0.63	2.23	0.62
Ser	0.35	1.85	0.59
Tyr	0.22	1.61	0.46
Total amino acids, %	7.44	43.06	10.67

### Nitrogen balance

Dietary treatment did not affect the initial or final body weight, daily feed intake, weight of feces and urine, or the ATTD of dry matter (Table 3). Daily nitrogen intake, nitrogen excretion in feces and urine, absorbed nitrogen, ATTD of nitrogen, and retained nitrogen (g/d) were greater (*P* < 0.05) for sows fed the high-protein diet compared with sows fed the low-protein diet. However, nitrogen retention as a percentage of intake and biological value were not different between treatments.

**Table 3:** Effects of dietary protein on apparent total tract digestibility (ATTD) of dry matter and nitrogen balance of gestating sows^1^

Item	Diet		SEM	*P-*value
	High protein	Low protein		
Initial body weight, kg	201.13	200.36	3.733	0.823
Final body weight, kg	209.36	208.30	3.810	0.765
Feed intake, kg/d	2.23	2.22	0.027	0.575
Dry feces output, g/d	0.21	0.21	0.005	0.237
ATTD of dry matter, %	89.93	90.10	0.215	0.515
Urine output, kg/d	14.22	12.86	1.540	0.491
Nitrogen intake, g/d	52.24	39.74	0.557	< 0.001
Nitrogen excretion in feces, g/d	6.77	6.05	0.198	0.001
Absorbed nitrogen, g/d	45.47	33.69	0.501	< 0.001
ATTD of nitrogen, %	87.04	84.79	0.386	< 0.001
Nitrogen excretion in urine, g/d	20.45	15.40	0.666	< 0.001
Retained nitrogen, g/d	24.96	18.28	0.764	< 0.001
Nitrogen retention, % of intake	47.79	45.85	1.422	0.338
Biological value^2^, %	54.82	54.15	1.702	0.771

^1^Data are least-squares means of 44 observations in the high-protein and 46 in the low-protein diet

^2^Calculated by dividing retained nitrogen by absorbed nitrogen and multiplying by 100 [18]

### Reproductive parameters

Differences in body weights of sows between treatment groups were not observed at breeding (Table 4), but sows and gilts fed the high-protein diet were heavier (*P* < 0.05) on d 104 compared with sows fed the low-protein diet, and no difference between treatments in the weight of sows at weaning was observed. The ADG tended to be greater (*P* < 0.10) for sows fed the high-protein diet compared with sows fed the low-protein diet during the gestation period, but no difference in ADG was observed during the lactation period. There was no difference between treatments in daily feed intake from breeding to d 104, from d 104 to farrowing or during the lactation period. The temperature of sows fed the low-protein diet was greater (*P* < 0.05) than that of sows fed the high-protein diets at the time of farrowing and 24 h later.

**Table 4:** Performance of sows fed high or low-protein diets during gestation and lactation^1^

Item	Diet		SEM	*P-*value
	High protein	Low protein		
Parity	1.68	1.66	0.197	0.938
Body weight, kg				
At breeding	191.47	188.18	3.054	0.448
Day 104 gestation	230.06	222.47	2.488	0.033
At 24 h after farrowing	213.42	208.05	2.402	0.116
At weaning	204.44	198.83	2.582	0.127
Average daily gain, kg				
Breeding to d 104 of gestation	0.368	0.327	0.016	0.072
Days 1 to 21 of lactation	−0.419	−0.437	0.069	0.836
Average daily feed intake, kg				
Breeding to d 104 of gestation	2.17	2.14	0.021	0.360
Pre-farrowing^2^	2.43	2.46	0.090	0.493
Days 1 to 21 of lactation	5.48	5.59	0.181	0.367
Estimated total milk yield^3^, kg	224.78	221.72	3.935	0.539
Estimated daily milk yield, kg	11.05	10.90	0.282	0.534
Temperature at farrowing, °C	38.12	38.34	0.150	0.046
Temperature 24 h after farrowing, °C	38.39	38.63	0.120	0.017

^1^Least squares means for each dependent variable represent 78 and 76 observations for the high-protein and low-protein diet, respectively

^2^Day 104 of gestation until farrowing

^3^Estimated milk yield was calculated as 4 g milk per 1 g of litter body weight gain [19]

The total number of pigs born per litter, total number of pigs born alive per litter, number of stillborn pigs per litter, and pigs per litter after cross-fostering were not different between the two treatments (Table 5), but sows fed the high-protein diet tended to produce fewer mummified pigs than sows fed the low-protein diet (*P* < 0.10). Live litter birth weight was not affected by diet, but the number of pigs with congenital deformities tended to be greater (*P* < 0.10) for sows fed the high-protein diet than for sows fed the low-protein diet. Litter weight after cross-fostering was greater (*P* < 0.05) for sows fed the high-protein diet than for sows fed the low-protein diet, but the individual pig weight after cross-fostering was less (*P* < 0.05) for sows fed the high-protein diet than for sows fed the low-protein diet. No differences between treatments were observed in litter ADG or individual pig ADG during lactation. Likewise, there was no difference in the number of pigs weaned per litter, in litter weaning weight, or in individual pig body weight at weaning. Pigs from sows fed the low-protein diet tended to have a greater (*P* < 0.10) survival rate after cross-fostering than pigs from sows fed the high-protein diet. Mortality due to crushing after cross-fostering tended to be greater (*P* < 0.10) for sows fed the high-protein diet than for sows fed the low-protein diet, but there was no effect of dietary treatment for the other causes of mortality after cross-fostering.

**Table 5:** Performance of litters from sows fed diets containing high or low protein during gestation and lactation^1^

Item	Diet		SEM	*P-*value
	High protein	Low protein		
Pigs per litter				
Total born	16.53	16.07	0.462	0.477
Born alive	15.61	14.83	0.446	0.220
After cross-fostering	13.60	13.00	0.418	0.315
Still born	0.83	0.91	0.119	0.574
Mummified	0.13	0.27	0.051	0.056
Weaned	12.68	12.37	0.405	0.594
Litter weight, kg				
Live at birth	20.53	19.49	0.447	0.103
After cross-fostering	19.00	18.12	0.304	0.041
At weaning	74.01	72.75	1.070	0.378
Litter average daily gain, kg	2.74	2.75	0.044	0.916
Individual pig weight, kg				
After cross-fostering	1.38	1.42	0.012	0.007
At weaning	5.84	5.88	0.074	0.655
Pig average daily gain, kg	0.22	0.22	0.003	0.440
Survival rate^2^, %	93.24	95.15	0.738	0.073
Pig mortality before cross-fostering, %^3^				
Crushed by sow	2.95	2.04	0.745	0.246
Low vitality	7.56	7.87	0.830	0.781
Rupture	0.33	0.09	0.127	0.241
Congenital deformity^4^	1.41	0.62	0.290	0.069
Pig mortality after cross-fostering, %^5^				
Crushed by sow/broken legs	2.69	1.50	0.463	0.069
Low vitality/starved/runt	2.62	2.31	0.509	0.642
Rupture^6^	1.24	0.91	0.322	0.475

^1^Least squares means for each dependent variable represent 78 and 76 observations for the high-protein and low-protein diet, respectively

^2^Survival rate was calculated as the number of pigs weaned divided by the number of pigs per litter after cross-fostering

^3^Calculated as the percentage of liveborn pigs that died before weaning, before cross-fostering

^4^Congenital deformities also include spraddle leg pigs and hernias

^5^Calculated as the percentage of live pigs after cross-fostering that died before weaning

^6^Include pigs found dead and pigs with swollen joints

### Milk composition and immune response

The concentration of fat and total solids in colostrum was not affected by diet (Table 6), but the concentration of fat, free fatty acids, protein, MUN, and lactose was greater (*P* < 0.05) in the milk collected on d 14 from sows fed the high-protein diets compared with milk from sows fed the low-protein diets. The IgG in milk and colostrum was also greater (*P* < 0.05) for sows fed the high-protein diet compared with sows fed the low-protein diet.

**Table 6:** Composition of milk samples collected on the day of farrowing and 14 d post-farrowing from sows fed diets containing high or low protein during gestation and lactation^1^

Item	Diet		SEM	*P-*value
	High protein	Low protein		
Colostrum				
Fat, %	5.12	4.80	0.255	0.265
Total solids, %	24.92	24.61	0.484	0.551
IgG^2^, mg/mL	81.07	75.47	1.094	< 0.001
Milk				
Fat, %	7.93	7.69	0.044	< 0.001
Free fatty acids, %	0.40	0.30	0.007	< 0.001
Protein, %	4.64	4.50	0.028	< 0.001
MUN^2^, mg/dL	45.54	42.36	0.194	< 0.001
Lactose, %	4.70	4.60	0.024	0.002
Other solids, %	6.24	6.25	0.017	0.772
Total solids, %	24.92	24.61	0.484	0.551
SCC^2^, 1,000/mL	98.00	100.53	1.812	0.254
IgG^2^, mg/mL	1.25	1.11	0.032	0.003

^1^Least squares means for each dependent variable represent 78 and 76 observations for the high-protein and low-protein diet, respectively

^2^*MUN* Milk urea nitrogen, *SCC* Somatic cell count, *IgG* Immunoglobulin G

Malondialdehyde was greater (*P* < 0.05) in serum from sows fed the low-protein diets compared with sows fed the high-protein diets, but white blood cell count and glutathione peroxidase were greater (*P* < 0.05) in serum from sows fed the high-protein diet than those fed the low-protein diet (Table 7). Most serum cytokines and white blood cell differential did not differ between treatments on d 14 of lactation, but IL-4 was greater (*P* < 0.05) in sows fed the high-protein diet than in sows fed the low-protein diet. Likewise, sows fed the high-protein diet tended to have greater (*P* < 0.10) concentration of IFN-γ compared with sows fed the low-protein diet, whereas sows fed the low-protein diet tended to have greater (*P* < 0.10) IL-2 in blood compared with sows fed the high-protein diet.

**Table 7:** Serum immune response of sows fed diets containing high or low protein during gestation and lactation^1,2^

Item	Diet		SEM	*P-*value
	High protein	Low protein		
Malonaldehyde, nmol/mL	2.77	2.89	0.042	0.026
Glutathione peroxidase, U/L	1,128.15	1,052.23	11.106	< 0.001
Leukocyte profile				
White blood cell count, 10^3^/μL	14.31	13.38	0.331	0.039
Neutrophils, %	50.99	50.70	1.208	0.868
Lymphocytes, %	36.57	36.16	0.956	0.757
Monocytes, %	5.95	6.11	0.312	0.715
Eosinophils, %	4.79	5.19	0.276	0.306
Basophils, %	1.06	0.96	0.100	0.458
Cytokines, ng/mL				
IFN-γ	18.03	13.50	2.131	0.071
IL-1α	0.21	0.21	0.026	0.905
IL-1β	0.64	0.83	0.120	0.118
IL-1Ra	1.58	1.44	0.181	0.542
IL-2	0.98	1.32	0.162	0.093
IL-4	15.15	9.66	1.574	0.005
IL-6	0.61	0.65	0.077	0.736
IL-8	0.06	0.08	0.013	0.461
IL-10	5.53	4.95	0.566	0.451
IL-12	0.65	0.71	0.099	0.524
IL-18	5.68	5.20	0.752	0.532
TNF-α	0.81	0.24	0.379	0.282

^1^Least squares means for each dependent variable represent 78 and 76 observations for the high-protein and low-protein diet, respectively, after the removal of outliers

^2^*IFN-γ* Interferon-gamma, *IL-* Interleukin-, *IL-1Ra* Interleukin-1 receptor antagonist, *TNF-α* Tumor necrosis factor-α

## Discussion

The analyzed values for crude protein and total AA in experimental diets for gestating and lactating sows were in close agreement with formulated values, which indicates correct mixing of diets. Likewise, the analyzed concentrations of nutrients in corn, SBM, and soybean hulls were consistent with reported data [12].

### Nitrogen balance

Dietary protein reduction in the low-protein diet was accomplished by reducing SBM and adding more corn and crystalline AA to ensure adequate digestible AA for gestating and lactating sows. The lack of an effect of reducing crude protein in the diet on feed intake in gestating sows was in agreement with reported data [20, 21]. The energy value of SBM is close to the energy value of corn [22, 23], and the observation that there were no differences in ATTD of DM between the high and low-protein diets was, therefore, expected. The ATTD of crude protein in corn is less than the ATTD of crude protein in SBM [24], and the reduction in the ATTD of nitrogen that was observed when SBM was replaced by corn and crystalline AA is, therefore, due to the lower crude protein digestibility in corn. This observation is in agreement with data from growing pigs fed diets where SBM was replaced by corn and crystalline AA [25, 26].

Soybean meal is used as the principal source of AA in swine diets in most pig-producing countries [12]. When SBM is included in corn-SBM diets to meet the requirements of indispensable AA, dietary concentrations of other AA often exceed the requirement [27]. The excess AA undergo deamination, resulting in nitrogen losses because of urinary excretion of nitrogen [28]. Reducing dietary protein by 3 percentage units in gestation diets in the present experiment decreased urinary nitrogen excretion by approximately 5 g/d, which agrees with data from sows fed low-protein diets [29, 30].

Both gestation diets were formulated to provide sufficient standardized ileal digestible indispensable AA to support fetal development and maternal growth. Therefore, similar nitrogen retention was expected between the diets. However, the observation that nitrogen retention, measured as g/d, was greater in sows fed the high-protein diet compared with sows fed the low-protein diet supplemented with crystalline AA indicates that crystalline AA were not used with the same efficiency as AA from SBM. This observation is in agreement with reported data [2, 20], and may be due to the faster rate of absorption and metabolism of crystalline AA compared with protein-bound AA [10], resulting in an imbalance in AA supply for the cells at the sites of protein synthesis [31]. This indicates that a limited inclusion of crystalline AA in low-protein diets is preferred because imbalances in AA supply may reduce nitrogen retention for fetal, mammary, and maternal tissue development [32]. Another possibility is that the reduced crude protein in the diets failed to meet the requirements for dispensable AA in gestating sows, which may have limited protein synthesis and nitrogen retention by sows fed the low-protein diet. However, both diets were formulated to meet or exceed requirements for digestible AA [12], and the analyzed proportion of indispensable AA to total AA in the diets averaged 45.56% and 46.79% corresponding to dispensable AA proportions of 54.44% and 53.21%, respectively. These values are within the range of what has been hypothesized for growing pigs to provide adequate nitrogen for the de novo synthesis of dispensable AA, and a similar proportion is also expected to be sufficient for sows [33]. Therefore, it is unlikely that there was a lack of nitrogen to synthesize dispensable AA in the low-protein diet. It is, however, possible that the nitrogen needed to maximize reproductive performance of sows is less than what is needed to maximize nitrogen retention, and if that is the case, the observed difference in nitrogen retention may not have implications for reproductive performance.

Nitrogen retention as a percentage of intake was approximately 46% for both diets, which is greater than values previously reported for gestating sows [20, 29]. The implication of this observation is that when formulating diets with a balanced AA profile, nitrogen retention as a percentage of intake is maximized. Formulating diets based on the basis of standardized ileal digestibility of AA also provides balanced diets, resulting in a greater retention of nitrogen, which will reduce AA deamination and, therefore, also reduce nitrogen excretion.

### Reproductive performance

During the gestation period, sows are usually restricted in feed intake according to the visual assessment of body condition to avoid problems associated with excessive body weight gain [34]. The observation that sows fed the low-protein diet had reduced body weight and ADG during the gestation period, even though no differences in feed intake between the two groups of sows were observed, indicates that although diets were balanced in AA and met or exceeded the requirements for digestible indispensable AA, sows fed the high-protein diet had greater whole body protein deposition due to the greater retention of nitrogen in the gestation period, which increased the weight at the end of the gestation period. This observation is in agreement with data from sows fed diets with different levels of crude protein [7, 35], which demonstrated that greater dietary protein in the diet can support greater maternal tissue growth during gestation. It is, however, also possible that the tendency for reduced ADG during gestation was due to reduced backfat deposition, but because backfat was not determined in sows in this experiment, we cannot confirm this hypothesis. The lack of difference between the two treatments for litter size at birth indicates that although sows fed the low-protein diet had lower nitrogen retention, and therefore reduced body weight in late gestation, sows prioritized protein for fetal development. The reduced nitrogen retention in sows fed the low-protein diet, therefore, likely is due to reduced retention of nitrogen in the body of sows.

During lactation, feed is offered to the sow on an ad libitum basis to meet the requirement of sows for milk component synthesis and to limit the mobilization of tissue reserves [36]. The lack of differences between treatments in feed intake, body weight at weaning, and ADG of the lactating sows is in agreement with results from other experiments with lactating sows fed diets with different inclusions of protein [2, 37], although greater body weight loss of sows during lactation has also been reported for sows fed low-protein diets [29]. Feeding high-protein diets to sows during gestation and lactation may reduce milk yield by sows fed organic diets [21], but the calculated milk yield of sows in this experiment was around 11 kg/d and did not differ between treatments, which is in agreement with other experiments where milk yield was between 10.5 and 13.8 kg/d [38–40].

In the net energy system, dietary starch and fat are assumed to be used more efficiently than protein because protein catabolism requires ATP for urea synthesis, protein turnover, and nitrogen excretion, which produces heat [41]. Consequently, reducing crude protein in the diet or adding fat is expected to reduce heat production [42, 43]. Although heat production was not measured in the current experiment, the rectal temperature of sows fed the high-protein diet was reduced compared with sows fed the low-protein diet, which does not support the assumption that high-protein diets increase body temperature in sows during farrowing or 24 h after farrowing. Heat production is positively correlated with energy supply in lactating sows, due to increased feed intake and milk synthesis [44, 45]). Therefore, reduced protein in the diet may have increased the metabolic heat of the sow due to an increase in body protein mobilization for milk protein synthesis, but because heat production was not measured in this experiment, we cannot confirm this hypothesis. It is also possible that the increased temperature is due to an intensified inflammatory response during parturition, but because we did not measure the inflammatory response during parturition, we cannot verify this speculation. It is also noted that sows on both treatments had body temperatures that were within the normal physiological range.

### Litter performance

The observation that the number of pigs born, the number of pigs weaned, litter weight at weaning, and litter ADG did not differ between sows fed the two diets is consistent with previous data indicating that nutrients for milk production are derived from both the diet and from body reserves [21, 38, 46], and sows have the ability to maintain milk production even when feed intake is reduced [47]. Milk yield is largely independent of feed intake unless body reserves are depleted, and a decrease in litter growth is more closely associated with energy restriction than with protein restriction [44, 45]. This may be the reason no differences in litter growth were observed between treatments in this experiment, which is also in agreement with data from sows fed low-protein diets [29, 37].

### Immunological measurements

Soy bioactive compounds (i.e., isoflavones and saponins) may enhance milk composition and the antioxidant status of the sow [48]. The reduced concentration of IgG in milk on d 14 compared with colostrum is consistent with data that report greater concentration of IgG in colostrum, but a decrease in concentration during lactation [49]. The observation that sows fed the high-protein diet had greater concentrations of IgG, protein, fat, and total solids than sows fed the low-protein diet, is in agreement with data that compared high and low-protein diets or different levels of soy isoflavone inclusion [40, 50]. Likewise, concentrations of lactose, fat, and protein in milk are also in agreement with reported data [40, 51, 52]. The lack of differences in individual pig body weight at weaning between treatments indicates that the improved quality of milk from sows fed the high-protein diet supported adequate growth, even though pigs from these sows had a lower BW after cross-fostering compared with pigs from sows fed the low-protein diet [53]. The implication of this observation is that although sows are capable of mobilizing body reserves to maintain milk quality, inclusion of greater quantities of SBM in the diet provides sufficient nutrients and immune support to compensate for the reduced BW of pigs.

Oxidative stress that animals undergo during gestation may influence the implantation and development of fetuses in the uterus during the early gestation stage [54, 55]. Soy bioactive compounds in SBM have also been classified as health-promoting due to their properties as anti-inflammatory agents and antioxidants [56]. Malondialdehyde has been used as a marker for lipid peroxidation in sows during lactation [57], and enzymatic antioxidants, such as glutathione peroxidase, are factors that reduce oxidative stress [58]. Lactation is characterized by an increase in metabolic activity associated with milk production, which may increase oxidative metabolism and reactive oxygen species production [59].

Although crude protein was reduced by only 3.5%, SBM inclusion in the low-protein diets was reduced by almost 50%, indicating that, in addition to the crude protein concentration, the protein source was also changed. Therefore, differences between the two diets may not only be due to the reduction of crude protein, but also to the reduced supply of soybean protein-associated compounds. During digestion, intact proteins may release different bioactive peptides, whereas crystalline AA provide only the free AA. Consequently, reduced SBM inclusion may have decreased the intake of soy bioactive compounds and peptides with potential antioxidant and anti-inflammatory effects. Therefore, the observed differences in concentrations of glutathione peroxidase and malondialdehyde between treatments during mid-lactation may reflect the reduced inclusion of soy bioactive compounds in the low-protein diet, which may affect the oxidative status of the sows. The implication of this observation is that soy bioactive compounds may improve the antioxidant-related defense of the sow during periods of high metabolic demand, such as lactation and gestation. However, values for malondialdehyde for both groups of sows during mid-lactation were between 2.77 and 2.89 nmol/mL, which are less than values reported for sows kept under thermoneutral conditions and fed diets containing more than 14% protein [59, 60]. This indicates that under the conditions of the current experiment, sows were not subjected to extreme oxidative stress. It is, however, acknowledged that there are other biomarkers for antioxidant status that could have been measured (i.e., total antioxidant capacity and superoxide dismutase). Likewise, to provide more robust data for the impact of diet SBM concentration on antioxidant status, and to confirm the data obtained in this experiment, it may be necessary to collect blood samples throughout gestation, at farrowing, and at several time points in lactation. Having multiple sampling points and analysis of more antioxidant markers would provide an opportunity to elucidate the dynamic impact of diets on antioxidant- and inflammatory status of sows during the entire reproductive period, which could not be provided from the current data where blood was collected only one time during lactation.

When an animal is exposed to different stimuli associated with inflammation and infection, innate immune cells, including white blood cells, release pro- or anti-inflammatory cytokines [61]. The functional definition of an anti-inflammatory cytokine is the ability to inhibit the synthesis of pro-inflammatory cytokines such as IL-1, IL-2, IL-12, IL-18, IFN-γ, and TNF-α. Therefore, the observation that sows fed the high-protein diet had greater concentrations of anti-inflammatory cytokines indicates that these sows had improved immunity, which may also be a result of the greater intake of bioactive compounds from SBM, which may have improved the immune system of sows. However, due to the design of the experiment it was not possible to separate effects of greater concentrations of protein and possible effects of bioactive compounds and future research is, therefore, needed to specifically address the hypothesis that bioactive compounds in SBM have beneficial effects on reproductive performance of sows. In addition, bioactive compounds were not measured in the diets used in the experiment or the samples collected from sows, which prevents strong conclusion relative to these compounds.

## Conclusions

Feeding low-protein diets supplemented with crystalline AA to gestating and lactating sows maintained overall reproductive performance, but reduced nitrogen utilization efficiency during gestation compared with sows fed high-protein diets based on only corn and SBM. The greater levels of SBM in high-protein diets also appeared to result in greater antioxidant-related indicators, immune function, and milk quality. These results indicate that maintaining an adequate level of SBM in diets for sows is essential to optimize nitrogen retention, antioxidant-related indicators, and milk composition in sows.

## Data Availability

The datasets used and/or analyzed during the current study are available from the corresponding author upon reasonable request.

## Abbreviations

AA: Amino acids
ADFI: Average daily feed intake
ADG: Average daily gain
ATTD: Apparent total tract digestibility
IFN-γ: Interferon-gamma
IgG: Immunoglobulin G
IL-: Interleukin-
MUN: Milk urea nitrogen
SBM: Soybean meal
SCC: Somatic cell count
TNF-α: Tumor necrosis factor-α
